# Similar but different: The partially redundant roles of tomato Pic3 and Pic12 in immunity

**DOI:** 10.1093/plphys/kiae441

**Published:** 2024-08-22

**Authors:** Yee-Shan Ku

**Affiliations:** Assistant Features Editor, Plant Physiology, American Society of Plant Biologists; School of Life Sciences and Centre for Soybean Research of the State Key Laboratory of Agrobiotechnology, The Chinese University of Hong Kong, Hong Kong SAR, China

Plant immunity, the process through which plants resist pathogens, impacts environmental health and food availability. From the initial pathogen invasion through the induction of defense responses, a series of events occurs involving the activation or inactivation of proteins in signaling networks. Type 2C protein phosphatases (PP2Cs) regulate various stress responses by dephosphorylating their target proteins (Liang and Zhang 2022). PP2Cs are large gene families in plants. For example, the model plant Arabidopsis has 76 *PP2C* genes (Kerk et al. 2002), and tomato has 92 *PP2C* genes (Qiu et al. 2022). The substrate specificity of PP2Cs determines their regulatory roles in different stress responses. Although PP2Cs have been implicated in regulating plant immunity, the full picture is far from complete.

A previous study in tomato reported the differential expressions of some *PP2C*s in response to PAMP (pathogen-associated molecular pattern) and pathogen treatments (Sobol et al. 2022). These PP2Cs were referred to as PP2C immunity candidates (Pics). In a new study by Xia et al., the authors generated *pic* mutants, tested their resistance to *Pseudomonas syringae* pv. *tomato*, and found that the single mutations of *Pic3* and *Pic12* promoted immunity (Xia et al. 2024). The highly similar proteins share 84% amino acid identity.

The authors then dissected the functional domains of Pic3 and Pic12 proteins. The S-palmitoylation site prediction tool GS-Palm revealed a palmitoylation motif at the N terminus of Pic3. While native Pic3 localizes at the cell periphery, the mutation of the palmitoylation site rendered the losses of the cell-membrane association property as well as the negative function in immunity. The mutation of the phosphatase catalytic domain of Pic3 also eliminated its negative effect on immunity. The corresponding mutations of Pic12 had the same effects.

The authors next investigated whether the 2 proteins act independently or redundantly. A detailed analysis showed that the mutation of Pic3 promoted immunity in older leaves but not in the younger ones, while the mutation of Pic12 promoted immunity in both older and younger leaves. In the older leaves, the double mutation of Pic3 and Pic12 further promoted immunity compared with the single mutations. However, in the younger leaves, the double mutation had a similar effect compared with the *pic12* single mutation (Fig.). The results suggested the redundancy of Pic3 and Pic12 in the older leaves. However, in the younger leaves, Pic12 acts independently of Pic3.

**Figure.**
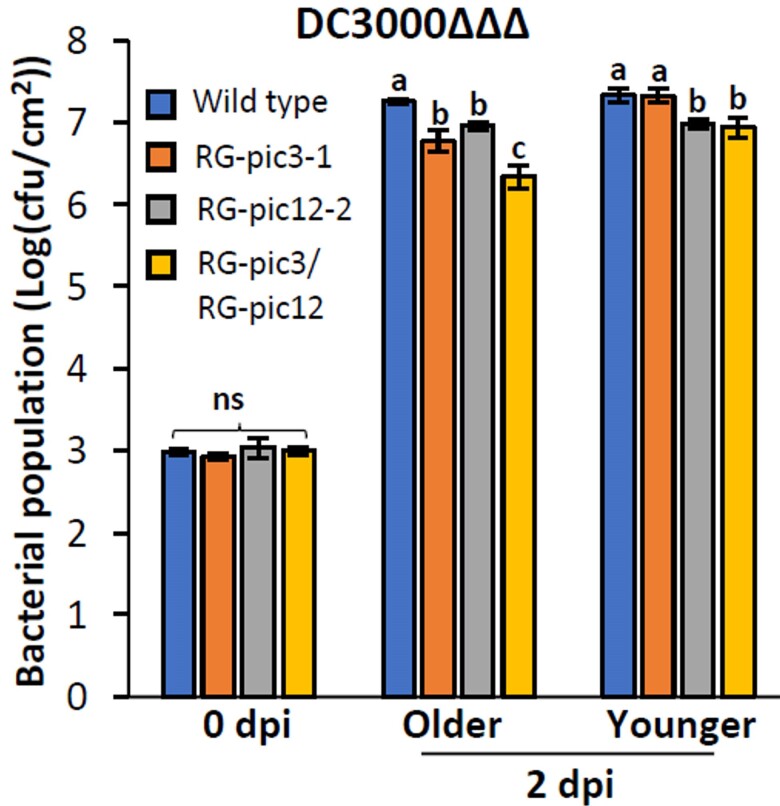
The single mutations of Pic3 and Pic12 (RG-pic3 and RG-pic12) supported smaller bacterial populations of *Pseudomonas syringae* pv. *tomato* strain DC3000ΔΔΔ in older leaflets of tomato plants through enhancing immunity. The double mutation of Pic3 and Pic12 (RG-pic3/RG-pic12) further promoted immunity compared with RG-pic3 and RG-pic12 in the older leaflets. In the younger leaflets, only RG-pic12 but not RG-pic3 had enhanced immunity. The figure is adapted from Xia et al. 2024 (Xia et al. 2024).

Using RNA-seq, the authors also investigated the differentially expressed genes (DEGs) between wild-type plants and *pic3* mutant plants under pathogen infection. In the *pic3* older leaves that had enhanced immunity, the DEGs were enriched in categories including defense responses, protein phosphorylation, and subcellular localization. The expression analysis results supported the above observation that the subcellular localization and phosphatase activity contribute to its negative role in immunity.

Based on amino acid sequences, PP2Cs in tomato are divided into 13 clades (Sobol et al. 2022). Based on the protein domain combinations, the proteins are classified into 17 structural groups (Sobol et al. 2022). Further structural and functional studies are needed to dissect their biological roles. Hinted by the expression responses of the *PP2C* genes, Xia et al. demonstrated strategies to delineate the functions of partially redundant homologous proteins by site-directed mutagenesis, loss-of-function in planta study, and high-throughput sequencing technology (Xia et al. 2024). In addition to enriching the understanding of PP2Cs in tomato, the study provides an example for functional studies of highly similar proteins.
